# Quad Element MIMO Antenna for C, X, Ku, and Ka-Band Applications

**DOI:** 10.3390/s23208563

**Published:** 2023-10-18

**Authors:** Raj Kumar Mistri, Santosh Kumar Mahto, Ajit Kumar Singh, Rashmi Sinha, Ahmed Jamal Abdullah Al-Gburi, Thamer A. H. Alghamdi, Moath Alathbah

**Affiliations:** 1 Department of Electronics and Communication Engineering, Indian Institute of Information Technology, Ranchi 834010, Jharkhand, India; raj.rtcit.kum@gmail.com (R.K.M.); ajitsingh31393@gmail.com (A.K.S.); 2 Department of Electronics and Communication Engineering, National Institute of Technology, Jamshedpur 831014, Jharkhand, India; 3 Center for Telecommunication Research & Innovation (CeTRI), Fakulti Teknologi dan Kejuruteraan Elektronik dan Komputer (FTKEK), Universiti Teknikal Malaysia Melaka (UTeM), Jalan Hang Tuah Jaya, Durian Tunggal, Melaka 76100, Malaysia; 4 Wolfson Centre for Magnetics, School of Engineering, Cardiff University, Cardiff CF24 3AA, UK; 5 Department of Electrical Engineering, College of Engineering, King Saud University, Riyadh 11451, Saudi Arabia

**Keywords:** channel capacity, channel capacity loss, diversity gain, mean effective gain, mm-wave, multiple-input and multiple-output, total active reflection coefficient

## Abstract

This article presents a quad-element MIMO antenna designed for multiband operation. The prototype of the design is fabricated and utilizes a vector network analyzer (VNA-AV3672D) to measure the S-parameters. The proposed antenna is capable of operating across three broad frequency bands: 3–15.5 GHz, encompassing the C band (4–8 GHz), X band (8–12.4 GHz), and a significant portion of the Ku band (12.4–15.5 GHz). Additionally, it covers two mm-wave bands, specifically 26.4–34.3 GHz and 36.1–48.9 GHz, which corresponds to 86% of the Ka-band (27–40 GHz). To enhance its performance, the design incorporates a partial ground plane and a top patch featuring a dual-sided reverse 3-stage stair and a straight stick symmetrically placed at the bottom. The introduction of a defected ground structure (DGS) on the ground plane serves to provide a wideband response. The DGS on the ground plane plays a crucial role in improving the electromagnetic interaction between the grounding surface and the top patch, contributing to the wideband characteristics of the antenna. The dimensions of the proposed MIMO antenna are 31.7 mm × 31.7 mm × 1.6 mm. Furthermore, the article delves into the assessment of various performance metrics related to antenna diversity, such as ECC, DG, TARC, MEG, CCL, and channel capacity, with corresponding values of 0.11, 8.87 dB, −6.6 dB, ±3 dB, 0.32 bits/sec/Hz, and 18.44 bits/sec/Hz, respectively. Additionally, the equivalent circuit analysis of the MIMO system is explored in the article. It’s worth noting that the measured results exhibit a strong level of agreement with the simulated results, indicating the reliability of the proposed design. The MIMO antenna’s ability to exhibit multiband response, good diversity performance, and consistent channel capacity across various frequency bands renders it highly suitable for integration into multi-band wireless devices. The developed MIMO system should be applicable on n77/n78/n79 5G NR (3.3–5 GHz); WLAN (4.9–5.725 GHz); Wi-Fi (5.15–5.85 GHz); LTE5537.5 (5.15–5.925 GHz); WiMAX (5.25–5.85 GHz); WLAN (5.725–5.875 GHz); long-distance radio telecommunication (4–8 GHz; C-band); satellite, radar, space communications and terrestrial broadband (8–12 GHz; X-band); and various satellite communications (27–40 GHz; Ka-band).

## 1. Introduction

Multiple input multiple output (MIMO) antenna technology is an indispensable option for wireless transmissions to meet more data needs and increase spectral efficiency. MIMO antenna configurations for 4G will also be an important system for enabling 5G [1]. This 5 G communication must promote two frequency bands: the sub-6 GHz band and the mm-wave band. Focused fast transmission, shorter latency, and increased bit rate are achievable in mm-wave bands due to spectrum commitment. However, mm-wave communication experiences congestion and loss. Therefore, there should be diversified integrated antenna architectures that can provide a good frequency band [2]. Implementation of these systems seems difficult due to antenna dimensions and less separation of antennas that generally operate in the microwave band [3]. The proposed MIMO design in [4] has four antennas out of which two elements are operating at 3.46 GHz and the other two at 17 GHz. In this design, no elements are operating in the mm-wave 5G band. In [5], a quad-element inverted F-shaped antenna and a planar-connected array with a dimension of 100 mm × 60 mm × 0.76 mm are presented. This design has been fabricated on an RO-4350 substrate, and the proposed structure has an operating frequency of 2.1 GHz and 12.5 GHz. In [6,7], multi-layer MIMO antenna systems have been introduced with three elements and five elements. The design covers the mm-wave 5G band, but both fail to cover 5G sub-6 GHz and WLAN bands. The literature [8] consists of an eight-element MIMO antenna system in which four elements operate in the 2.4–5.6 GHz band and the other four in the 23–30 GHz frequency band. In this design, the defected ground structure is implemented to lessen the mutual coupling among elements. In [9], an eight-element integrated MIMO antenna for sub-6 GHz 5G band and mm-wave band has been proposed. Out of the eight elements, four elements operate in the sub-6 GHz 5G band (i.e., 2.38–4.13 GHz and 5.04–6.12 GHz), and the other four elements operate in the mm-wave band (i.e., 22.28–29.28 GHz). The antenna design presented in [10,11,12] works in both the sub-6 GHz 5G band and mm-wave band; however, they do not cover the WLAN/Wi-Fi band. A miniaturized four-port MIMO antenna system has been developed in [13] for sub-6 GHz 5G band and WLAN applications. The introduced design has been operating at frequencies 2.6, 3.5, 4.8, and 5.8 GHz. A dual-band dual-port high isolation trident-shaped MIMO antenna system with an operating frequency band from 2.99 to 3.61 GHz and 4.53 to 4.92 GHz has been proposed in [14], which is operating in the sub-6 GHz 5G band only and doesn’t cover the mm-wave band. The literature reported in [15,16] operated a dual frequency band under the sub-6 GHz band and the operating frequency was 3.4–3.6, 4.76–5.04, and 3.95–4.04 GHz, respectively. On the other end, the literature reported in [17,18,19] only covers the mm-wave 5G band, which is a bandwidth of 1.23 GHz at 28 GHz and 1.06 GHz at 38 GHz, 26–30 GHz, 36–41.5 GHz, 26.65–29.2 GHz, and 36.95–39.05 GHz, respectively. Additionally, the literature [20] consists of a flower-shaped radiating patch where two rectangular-shaped slots in the ground plane provide a wide band of 2.83–14.0 GHz. In [21], a lotus-shaped radiating element with DGS in-ground and the addition of slits provides a 5G: n77/n78 wireless band with higher isolation (i.e., >16 dB).

This article consists of three sections. The first section covers the details of antenna geometry. The second section covers the detailed analysis of different diversity characteristics parameters like ECC, DG, $\left|MEG_{i}-MEG_{j}\right|$, TARC, CCL, and CC. The operating bands investigated by this structure are 3–15.5 GHz, 26.4–34.3 GHz, and 36.1–48.9 GHz, which are referred to as OFB1, OFB2, and OFB3, respectively. The minimum port isolation and ECC over the bands OFB1, OFB2, and OFB3 are observed as (15.1 dB, 0.11), (22 dB, 0.035), and (22.37 dB, 0.04), respectively. The third section provides a detailed analysis of simulated time-domain analysis in two different orientations, which are face to face (FTF) and side by side (STS). The fourth section covers the conclusion. In this study, all the simulation results are obtained from CST studio suite 2021. Computational work was conducted by MATLAB R2015a and the analysis and plotting by Origin 8.5 Pro. In all operating bands, the measured values provide good agreement with respect to the simulations.

## 2. Antenna Geometry

The proposed antenna element has a dual-side three-stage stair and a straight stick on the top of the stair. The partial ground plane has a small rectangle microstrip line just beneath the feed line. The introduction of a DGS on the ground plane serves to provide a wideband response. The DGS on the ground plane plays a crucial role in improving the electromagnetic interaction between the grounding surface and the top patch, contributing to the wideband characteristics of the antenna [22]. This DGS firstly functions as an extender of the antenna’s current path, thereby mitigating the resonant frequency of the antenna at its fundamental mode. Secondly, it acts as a suppressor of surface currents, thereby ameliorating isolation within the frequency bands spanning OFB1, OFB2, and OFB3. The proposed DGS approach maintains a diminished influence on the antenna’s intrinsic impedance characteristics, primarily since the supplementary current pathways are realized through the judicious etching of coupling slots on the ground plane. In this design, the straight stick is a 50-ohm microstrip feed line. The design is fabricated on an FR4 substrate having a thickness of 1.6 mm with a dielectric constant of 4.4, a loss tangent of 0.02, and a copper layer thickness of 35 um. 

**Figure 1:**
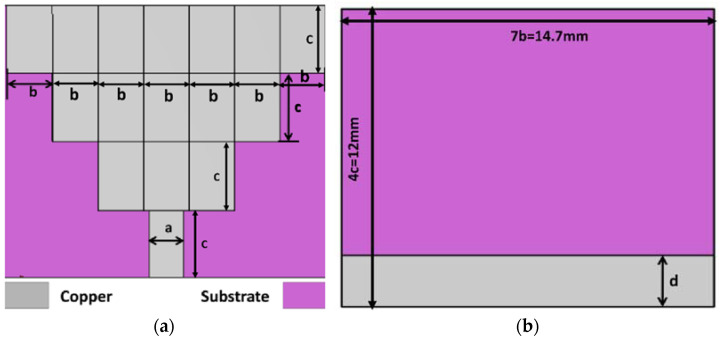

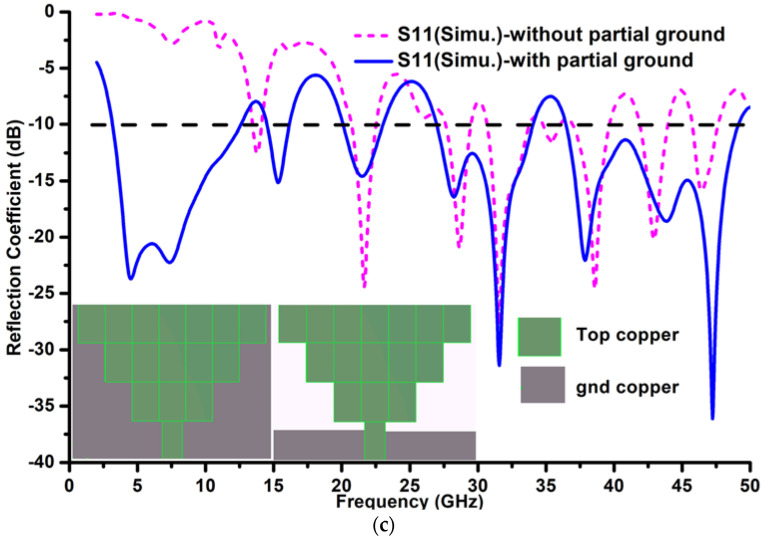
Single element antenna geometry: (**a**) Top view, (**b**) Bottom view, and (**c**) Simulates refle-tion coefficient.

The size of the single-element antenna is 14.7 mm × 12 mm × 1.6 mm. In this design, the following design variables are considered, which are “a”, “b”, “c” and “d”. For optimized performance of the antenna, the design variables a, b, c, and d are considered as 1.5 mm, 2.1 mm, 3 mm, and 2.3 mm, respectively. The top and bottom views of the proposed antenna element are shown in Figure 1a and Figure 1b, respectively. The simulated reflection coefficient is depicted in Figure 1c, where the antenna structure with a partial ground plane provides an excellent result in comparison to a structure with a full ground plane.

**Figure 2:**
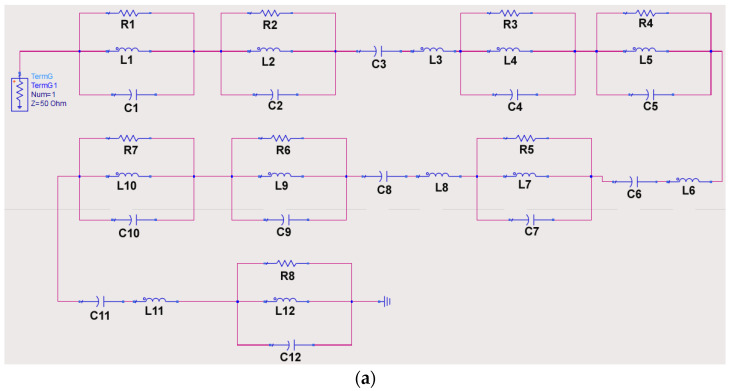

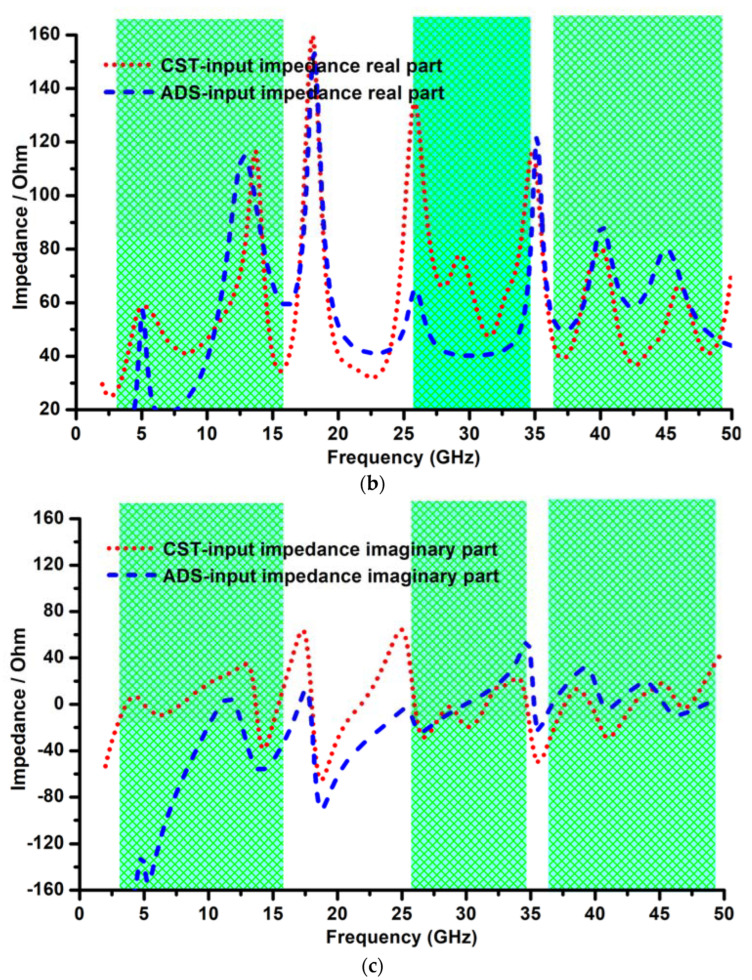
Proposed single antenna element: (**a**) Equivalent lumped circuit model (**b**) Impedance real part, and (**c**) Impedance imaginary part.

Furthermore, the conceptual equivalent circuit model for the proposed antenna was developed (Figure 2a). The initial approximate values for the lumped components were defined from the CST simulated antenna input impedance (Zin), and the equivalent lumped circuit parameters were then optimized using Keysight advanced design system (ADS) simulations [23]. In this article, eight RLC tank circuits are connected in series due to having eight adjacent resonant frequencies overlapping with each other (Figure 1c). Note that the addition of the series capacitor Cn and inductor Ln to the RLC circuits is necessary to correctly produce the imaginary part of the antenna input impedance [24,25]. 

The real (resistive part) and imaginary (reactance part) parts of the input impedance of the proposed MIMO antenna system are depicted in Figure 2. Figure 2b represents the real part of input impedance, obtained from CST and ADS simulation, whereas Figure 2c represents the imaginary part. Here, the proposed antenna shows good matching with the coaxial cable within all operating frequency bands.

The optimized values of lumped components are shown in Table 1.

The developed MIMO system should be applicable on n77/n78/n79 5G NR (3.3–5 GHz); WLAN (4.9–5.725 GHz); Wi-Fi (5.15–5.85 GHz); LTE5537.5 (5.15–5.925 GHz); WiMAX (5.25–5.85 GHz); WLAN (5.725–5.875 GHz); long-distance radio telecommunication (4–8 GHz; C-band); satellite, radar, space communications and terrestrial broadband (8–12 GHz; X-band); and various satellite communications (27–40 GHz; Ka-band). As mm-wave has a high carrier frequency, the signal baud rate could carry more data, resulting in a high data rate (bits/s/Hz) and a reduction in the buffering time in delivering high-quality videos [26]. The mm-wave has a limited range, this helps in cellular communication to expand the network coverage with the concept of frequency re-use through cell splitting and sectoring. The other applications of mm-wave are in radar and image sensing [27,28], next-gen Wi-Fi (WiGig), virtual reality for 3D rendering, medical applications [29,30], military applications [31,32], and IoT applications [33].

The developed MIMO antenna covers a 37–40 GHz frequency band, which has been reported in [34,35,36]. This also covers the FR2 bands above 6 GHz for 5G applications, which are n258/n259/n261 (24.25–29.5 GHz) and n260 (37–43.5 GHz) [37,38,39,40,41,42,43,44].

### 2.1. Antenna Operating Principle

**Figure 3:**
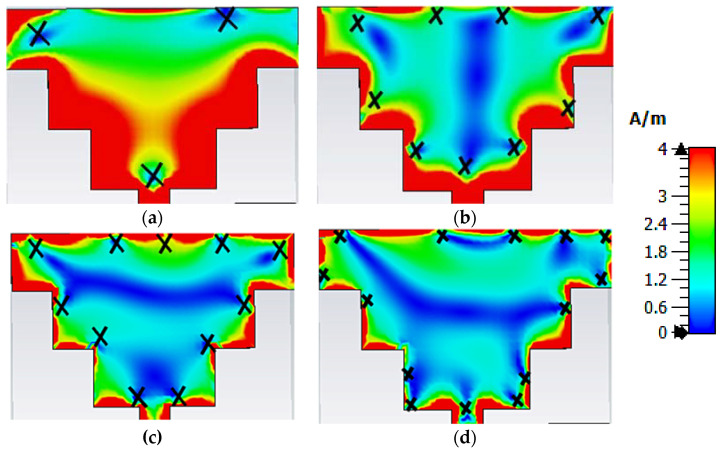
Surface current distributions on patch @ (**a**) 11.1 GHz, (**b**) 31.6 GHz, (**c**) 37.8 GHz, and (**d**) 46.7 GHz.

The interfacial current distributions at a frequency of 11.1 GHz, 31.6 GHz, 37.8 GHz, and 46.7 GHz are illustrated in Figure 3a, Figure 3b, Figure 3c and Figure 3d respectively. In all cases, the minimum surface currents are observed over the edges of the patch with a null point (cross-marked i.e., ‘X’). In Figure 3a–d the null points near the perimeter of the patch are found as 3, 9, 11, and 14, respectively. In order to find the resonance frequency, the number of null points plays a vital role.

Considering the ‘m’ number of null points that exist over the perimeter of the patch, then the guided wavelength ($\lambda_{g}$) can be expressed as

(1) $$\lambda_{g}=\frac{L}{m}\;$$

where *L* is the perimeter of the patch. In this article, L=10*d+6*c, which will be calculated as 47.4 mm. The resonance frequency can be calculated using Equation (2).

(2) $$f_{r}=\frac{V_{l}}{2L_{eff}\sqrt{\in_{eff}}}\;$$

where $V_{l}$ is the velocity of light, $L_{eff}$ is the effective length, and $\in_{eff}$ is effective permittivity. These values can be determined using Equations (3) and (4).

(3) $$L_{eff}=\frac{\lambda_{g}}{2}=\frac{L}{2*m}\;$$

(4) $$\in_{eff}=\frac{\in_{r}+1}{2}+\frac{\in_{r}-1}{2}\left\{{\left(1+12\frac{h}{W_{f}}\right)}^{-0.5}+0.04{\left(1-\frac{h}{W_{f}}\right)}^{-0.5}\right\}\;\;\;$$

where, $W_{f}$ is feed line width, h is substrate thickness, and $\epsilon_{r}$ is the dielectric constant of the substrate. For m = 3, 9, 11, and 14, the resonance frequency can be determined using Equation (2), and the obtained values are 10.4 GHz, 31.2 GHz, 38.1 GHz, and 48.5 GHz, respectively, which are nearer to the simulated resonance frequency. 

### 2.2. Proposed MIMO Antenna

Embedding multiple antennas into the same antenna system is seen as a promising solution, which can improve both the system’s channel capacity and the communication link’s quality. The MIMO systems provide a technique in which multiple independent channels send and receive data concurrently in the same radio channel. In the MIMO system, antennas at each end of the communications circuit are combined to minimize errors, optimize data speed, and improve the capacity of radio transmissions by enabling data to travel over many signal paths at the same time. It also improves the radio link capacity to attain the multipath propagation.

**Figure 4:**
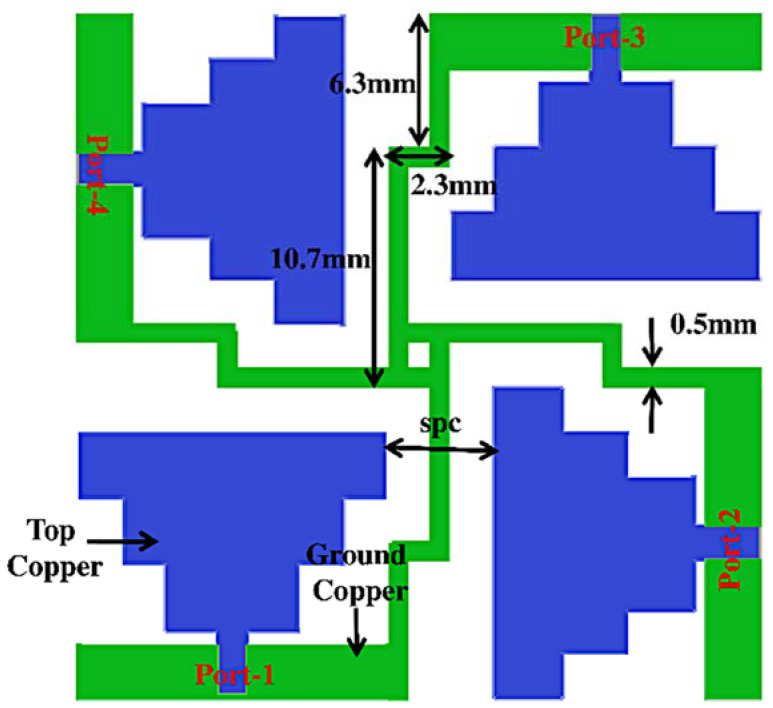
Proposed MIMO antenna system.

The proposed quad-element MIMO antenna system is illustrated in Figure 4. The size of MIMO is 31.7 mm × 31.7 mm. In this MIMO system, the port direction of any one of the antenna is kept perpendicular to every other port direction, and the placement of antenna elements is also symmetrical to one another. Both the properties’ symmetrical arrangement of elements and the orthogonality of ports lead to providing low ECC and better port isolation. Additionally, to achieve the common ground plane of the proposed MIMO system, four satire-step microstrip lines originating from each end of the rectangular microstrip line, which is present just beneath the ports in the ground plane, are connected to one common square strip. The creation of a common ground plane in this article is responsible for improving capacitance and inductance, leading to improved efficiency and gain of the MIMO antenna system. The design variable “spc” indicates the distance between two adjacent patches. In this article, “spc” is considered as 5 mm for the minimum isolation of 15 dB in the OFB1 band. 

**Figure 5:**
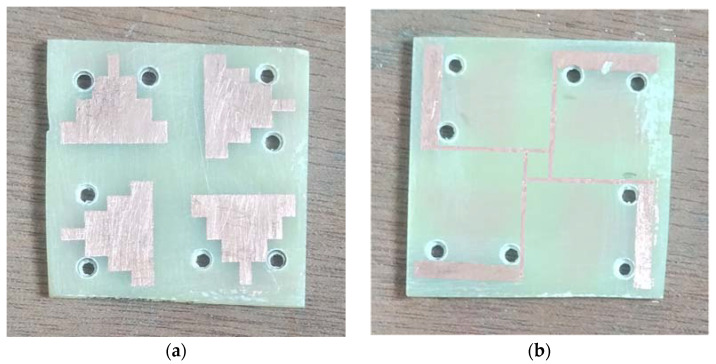
Fabricated antenna: (**a**) top view and (**b**) bottom view.

The fabricated MIMO system top and bottom views are depicted in Figure 5a and Figure 5b, respectively. 

## 3. MIMO Performance of the Proposed Antenna

**Figure 6:**
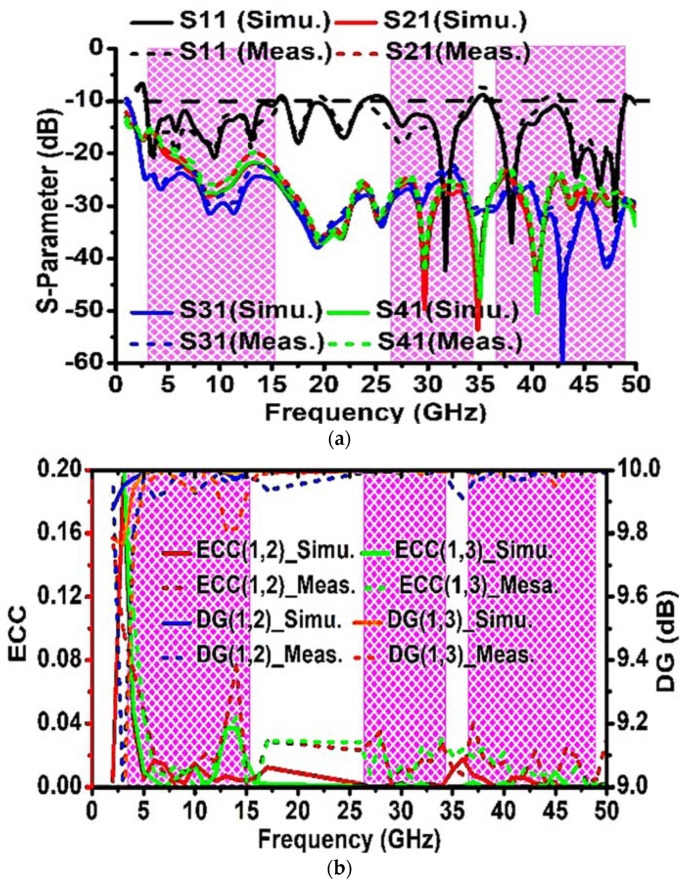
(**a**) S-parameter (simulated and measured), (**b**) ECC and DG (simulated and measured).

The MIMO performance of the proposed antenna system is investigated in terms of S-parameter, ECC, DG, radiation pattern, efficiency, gain, MEG, TARC, CCL, and CC. The measured −10 dB impedance bandwidth of the proposed MIMO antenna system are 3–15.5 GHz (% bandwidth of 116.27), 26.4–34.3 GHz (% bandwidth of 32.45), and 36.1–48.9 GHz (% bandwidth of 32.74). Over the entire frequency bands in OFB1, OFB2, and OFB3 the simulated minimum values of port isolation are 15.7 dB, 23.2 dB, and 23.2 dB, respectively; however, the measured minimum values of port isolation are 15 dB, 22 dB, and 22.3 dB, respectively. The simulated and measured S-parameter is depicted in Figure 6a. The envelope correlation coefficient ($\rho_{i,j}$) can be used for assessing the diversity efficiency of the MIMO antenna system.

The value of the ECC that is closest to zero implies that the MIMO antenna system has strong isolation and excellent diversity gain. Equation (5) [45] is used to calculate the measured ECC for a MIMO antenna system in this work.

(5) $$ECC=\rho_{i,j}=\frac{{\left|\iint_{4\pi }^{\;}\left[{\vec{M}}_{i}\left(\theta ,\varphi \right)\cdot {\vec{M}}_{j}\left(\theta ,\varphi \right)\right]dΩ\right|}^{2}}{\left[\iint_{4\pi }^{\;}{\left|{\vec{M}}_{i}\left(\theta ,\varphi \right)\right|}^{2}dΩ\right]\cdot \left[\iint_{4\pi }^{\;}{\left|{\vec{M}}_{j}\left(\theta ,\varphi \right)\right|}^{2}dΩ\right]}\;\;$$

where, ${\vec{M}}_{i}\left(\theta ,\varphi \right)$ indicates the 3D radiation pattern when antenna ‘*i*’ is excited, and ${\vec{M}}_{j}\left(\theta ,\varphi \right)$ indicates the 3D radiation pattern when antenna ‘*j*’ is excited. The solid angle is denoted by the symbol Ω. 

The degree of the improvement of the MIMO system is determined by diversity gain (DG). ECC can acquire the DG [46] calculation using Equation (6).

(6) $$DG_{i,j}=10\sqrt{1-ECC_{i,j}}$$

The simulated and measured ECC and DG over the frequency bands OFB1, OFB2, and OFB3 are depicted in Figure 6b. 

The simulated ECC and DG over the frequency bands OFB1, OFB2, and OFB3 are better than (0.213, 8.87 dB), (0.002, 9.89 dB), and (0.017, 9.91 dB), respectively, whereas the measured ECC and DG over the frequency bands OFB1, OFB2, and OFB3 are better than (0.1, 7.86 dB), (0.034, 9.97 dB), (0.04, 9.82 dB), respectively.

**Figure 7:**
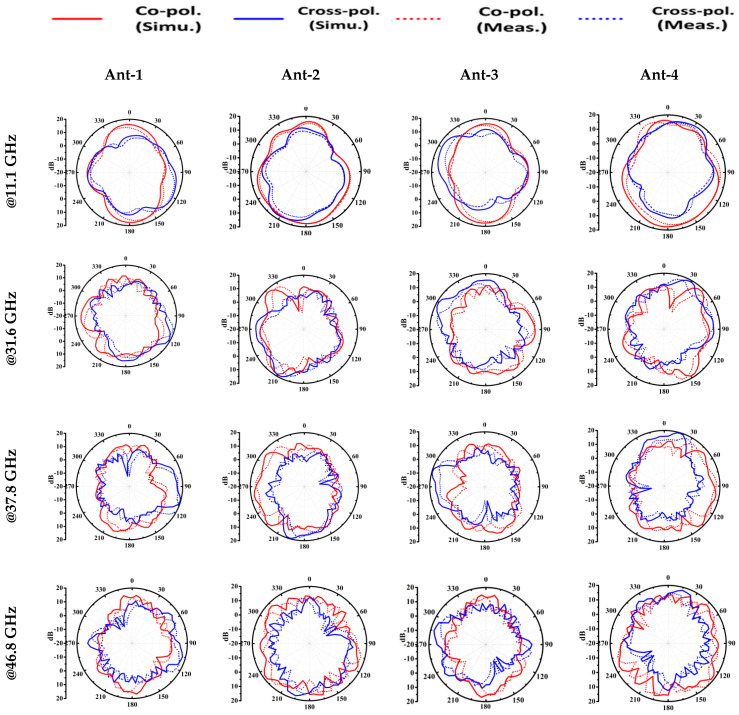
Radiation pattern of antennas @11.1 GHz, @31.6 GHz, @37.8 GHz, and @46.8 GHz in *XZ* plane.

Figure 7 depicts the simulated and measured two-dimensional (2-D) radiation patterns of Ant1-Ant4 at the frequencies 11.1 GHz, 31.6 GHz, 37.8 GHz, and 46.8 GHz in the *XZ* plane. 

At the frequency 11.1 GHz, 31.6 GHz, 37.8 GHz, and 46.7 GHz, the co-polar major lobe direction of Ant1-Ant4 are $(184^{0},\;183^{0},\;176^{0},\;177^{0}),\;(93^{0},\;148^{0},\;266^{0},\;212^{0}),$ $\left(162^{0},\;136^{0},\;198^{0},\;224^{0}\right)$, and $\left(185^{0},\;128^{0},\;175^{0},\;232^{0}\right)$, respectively. 

**Figure 8:**
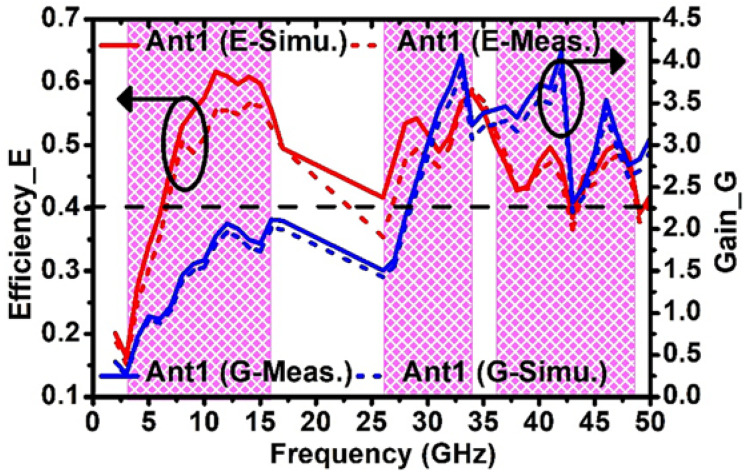
Simulated and measured gain-efficiency versus frequency plot.

Based on the observations, it is discovered that the radiation patterns of (Ant1, Ant3) and (Ant2, Ant4) are complementary to each other in the *XZ* plane. Such complementary radiation pattern qualities enable a low value of ECC between antennas and are also useful in producing self-isolated MIMO antenna systems. In Figure 8, the total efficiency and gain of Ant1 is illustrated. The simulated average efficiency and gain of Ant1 over the frequency bands OFB1, OFB2, and OFB3 are (49.1%, 1.76 dB), (58.2%, 4.65 dB), and (50.5%, 5.07 dB), respectively, whereas the measured average efficiency and gain over the frequency bands OFB1, OFB2, and OFB3 are (44.3%, 1.54 dB), (48.1%, 4.42 dB), and (44.9%, 4.85 dB), respectively. 

The mean effective gain (MEG) is a key measure for characterizing the performance of MIMO diversity. It compares the power received by the isotropic antenna to the power gained by the diversity antenna in a fade environment. The MEG of $i^{th}$ element of the MIMO system can be evaluated using Equation (7) [47].

(7) $$MEG_{i}=0.5\left[1-\sum_{n=1}^{N}{\left|S_{in}\right|}^{2}\right]$$

where N is the number of elements in the MIMO system. 

**Figure 9:**
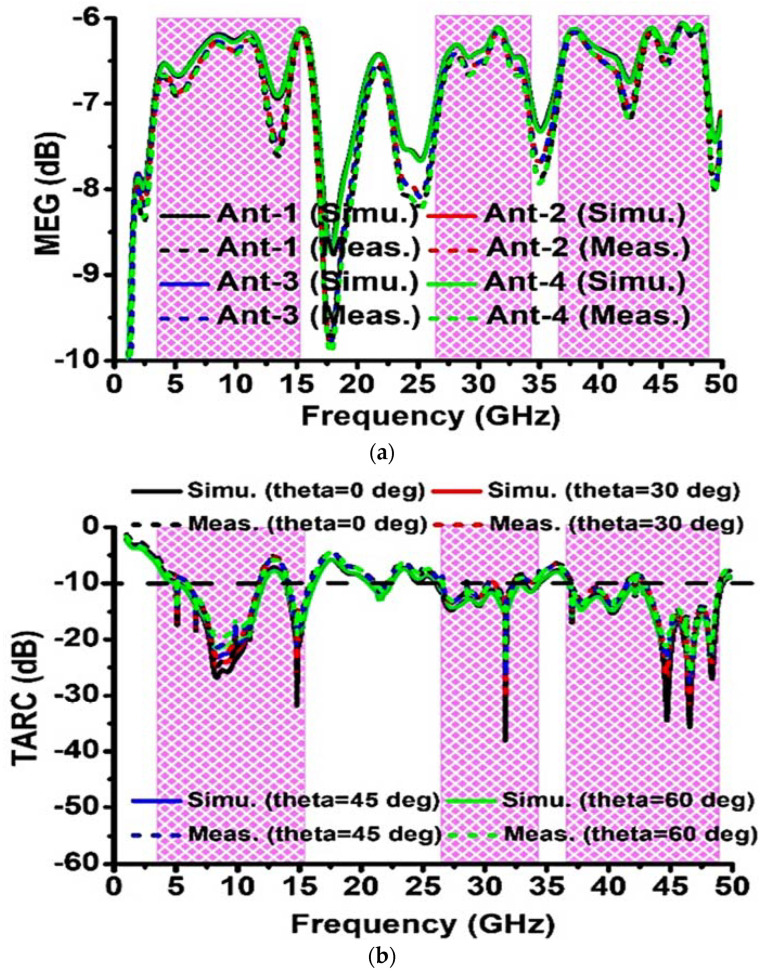
Proposed MIMO system (simulated and measured): (**a**) MEG and (**b**) TARC.

The typical value of $\left|MEG_{i}-MEG_{j}\right|$ should be less than 3 dB for a good MIMO antenna. The simulated and measured values of $MEG_{i}$ over the frequency bands OFB1, OFB2, and OFB3 are depicted in Figure 9a. The value of $\left|MEG_{i}-MEG_{j}\right|$ for distinct values of i and j are computed, where i≠j, 1≤i≤4 and 1≤j≤4. The maximum simulated values of $\left|MEG_{i}-MEG_{j}\right|$ over the frequency bands OFB1, OFB2, and OFB3 are 0.05 dB, 0.03 dB, and 0.082 dB, respectively; however, the maximum measured values of $\left|MEG_{i}-MEG_{j}\right|$ over the frequency bands OFB1, OFB2, and OFB3 are 0.16 dB, 0.25 dB, and 0.2 dB, respectively, which promises a good MIMO antenna in investigated operating bands. 

TARC is characterized as the square root of the overall reflected power divided by overall incident power. TARC provides a valid estimation of MIMO antenna efficiency since it contains information about the mutual coupling effect. The TARC for the N unit MIMO antenna setup can be computed using Equation (8) [48].

(8) $$TARC=N^{-0.5}\sqrt{\sum_{i=1}^{N}\;{\left|\sum_{K=1}^{N}S_{ik}e^{j\theta_{k-1}}\right|}^{2}}$$

The value of TARC is calculated with the assumption of $\theta_{k-1}=0^{0},\;30^{0},\;45^{0},\;60^{0}\;\left(\mathrm{where}\;1\leq k\leq 4\;\mathrm{and}\;k\;\mathrm{is}\;\mathrm{an}\;\mathrm{integer}\right)$. For a better performance in investigated bands, the TARC should be from 0 to 1 and the ideal value of TARC should be less than −10 dB. In this article, the simulated and measured TARC at $\theta_{k-1}=0^{0},\;30^{0},\;45^{0},\;60^{0}$ over the frequency ranges OFB1, OFB2, and OFB3 are below or about −10 dB except for the frequency band 12–14 GHz (in this band average, TARC is −6.9 dB). The simulated and measured TARC values are illustrated in Figure 9b. 

**Figure 10:**
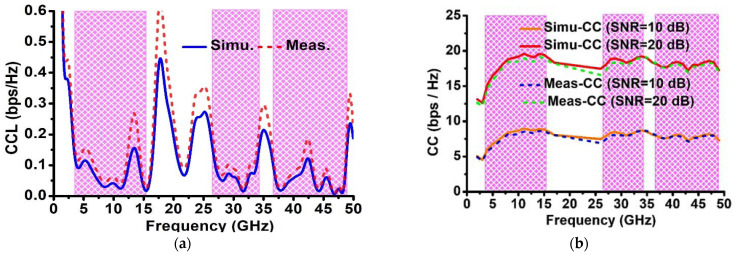
MIMO antenna (simulated and measured): (**a**) CCL and (**b**) CC.

An additional fundamental diversity attribute measure that may be used to assess MIMO performance is CCL, which shows the greatest rate at which data can be delivered without experiencing significant losses. Equation (9) [49,50] can be utilized to calculate CCL. The CCL ought to be below 0.4 bps/Hz throughout the operating frequency spectrum for the best MIMO antenna. Here, the greatest CCL (simulated and measured) value over the frequency ranges OFB1, OFB2, and OFB3 is less than 0.3 bps/Hz, indicating a strong candidate for use on the desired band. Figure 10a shows a CCL versus frequency plot.

(9) $$CCL=-\log_{2}\left[det\left(\varphi^{R}\right)\right]$$

$$where\;\varphi^{R}=\left(\begin{array}{cc} \begin{array}{cc} \alpha 11 & \alpha 12\\ \alpha 21 & \alpha 22 \end{array} & \begin{array}{cc} . & \begin{array}{cc} . & \alpha 1N \end{array}\\ . & \begin{array}{cc} . & \alpha 2N \end{array} \end{array}\\ \begin{array}{cc} . & .\\ \begin{array}{c} .\\ \alpha N1 \end{array} & \begin{array}{c} .\\ \alpha N2 \end{array} \end{array} & \begin{array}{cc} . & \begin{array}{cc} . & . \end{array}\\ \begin{array}{c} .\\ . \end{array} & \begin{array}{c} \begin{array}{cc} . & . \end{array}\\ \begin{array}{cc} . & \alpha NN \end{array} \end{array} \end{array} \end{array}\right),\left(\alpha ii=1-\left(\left|\sum_{j=1}^{j=N}S_{ij}^{*}S_{ji}\right|\right)\right)\;,$$

$$\left(\alpha ik=-\left|\overset{j=N}{\underset{j=1}{\sum }}S_{ij}^{*}S_{jk}\right|\right),1\leq i\leq N,1\leq k\leq N\;and\;N=4$$

Ergodic channel capacity (CC), which is determined under the presumption of zero ECC among the transmitting antennas, is the most efficient diversity characteristics metric for a MIMO system. Equation (10) is used to evaluate the channel matrix “H”.

(10) $$H=\sqrt{\rho_{scale,RX}}\;H_{iid}\sqrt{\rho_{scale,TX}}$$

where $H_{iid}$ is a 4 × 4 matrix whose elemental values are independent and identically distributed complex Gaussian random variables, $\rho_{scale,TX}$ is the calculated 4 × 4 matrix at the transmitter end with the consideration of null ECC and 100 percent efficiency, and $\rho_{scale,RX}$ is the calculated 4 × 4 matrix by employing measured ECC and efficiency as explained in [51].

Further the $\rho_{\mathrm{scale},\mathrm{RX}}$ is calculated using Equation (11).

(11) $$\rho_{scale,RX}=\sqrt{n_{total}}\rho_{RX}\sqrt{n_{total}}$$

The variables used in Equation (11) are $\rho_{RX}$ and $n_{total}$, which represents ECC and total efficiencies of receiving antennas. In order to estimate the ergodic channel capacity C, Equation (12) [52,53] is used.

(12) $$C=E\left\{log_{2}\left[det\left(I+\frac{SNR}{n_{T}}HH^{H}\right)\right]\right\}$$

Here, the variables in Equation (12), $H^{H}$, SNR, $n_{T}$, E and I, characterize Hermitian transpose, signal-to-noise ratio, number of antennas at the transmitter side, expectation in regard to different channel realizations, and 4 × 4 identity matrix, respectively. In this article, the ideal value of CC is computed using an average of more than 10,000 Rayleigh fading realizations with a 10 dB/20 dB SNR, null ECCs, and full efficiency of antennas. 

The ideal CC for 4 × 4 MIMO and 2 × 2 MIMO are 22.2 bps/Hz and 11.3 bps/Hz at SNR = 20 dB, respectively; whereas, for 4 × 4 MIMO and 2 × 2 MIMO, the ideal CC is 10.94 bps/Hz and 5.53 bps/Hz at SNR = 10 dB, respectively. The CC should be at least 60% of the ideal value for a MIMO antenna to work well. The simulated CC over the operating frequency bands OFB1, OFB2, and OFB3 is 4.53–8.95 bps/Hz, 7.46–8.70 bps/Hz, and 7.20–8.18 bps/Hz, respectively, at SNR = 10 dB, whereas the CC at SNR = 20 dB is 12.60–19.57 bps/Hz, 17.46–19.21 bps/Hz, and 17.20–18.56 bps/Hz, respectively. The measured CC over the operating frequency bands OFB1, OFB2, and OFB3 is 4.35–8.62 bps/Hz, 6.89–8.68 bps/Hz, 7.02–8.01 bps/Hz, respectively, at SNR = 10 dB; whereas, the CC at SNR = 20 dB is 12.13–19.13 bps/Hz, 16.55–19.18 bps/Hz, and 16.92–18.44 bps/Hz, respectively. The channel capacity versus frequency plot is depicted in Figure 10b. 

## 4. Simulated Time-Domain Analysis

**Figure 11:**
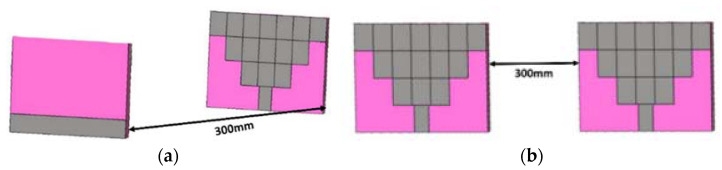
Simulated time-domain analysis in the layout: (**a**) Face to Face and (**b**) Side to Side.

Since the proposed antenna is wideband in nature, it is important to check its time domain characterization. Important time domain characteristics like group delay, phase response, and isolation analysis by keeping the antenna in face-to-face (FTF) and side-to-side (STS) orientation are studied. The two similar antennas are placed in two different configurations maintaining 300 mm, which are depicted in Figure 11. 

**Figure 12:**
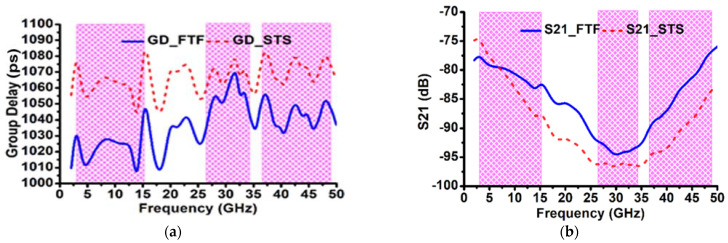

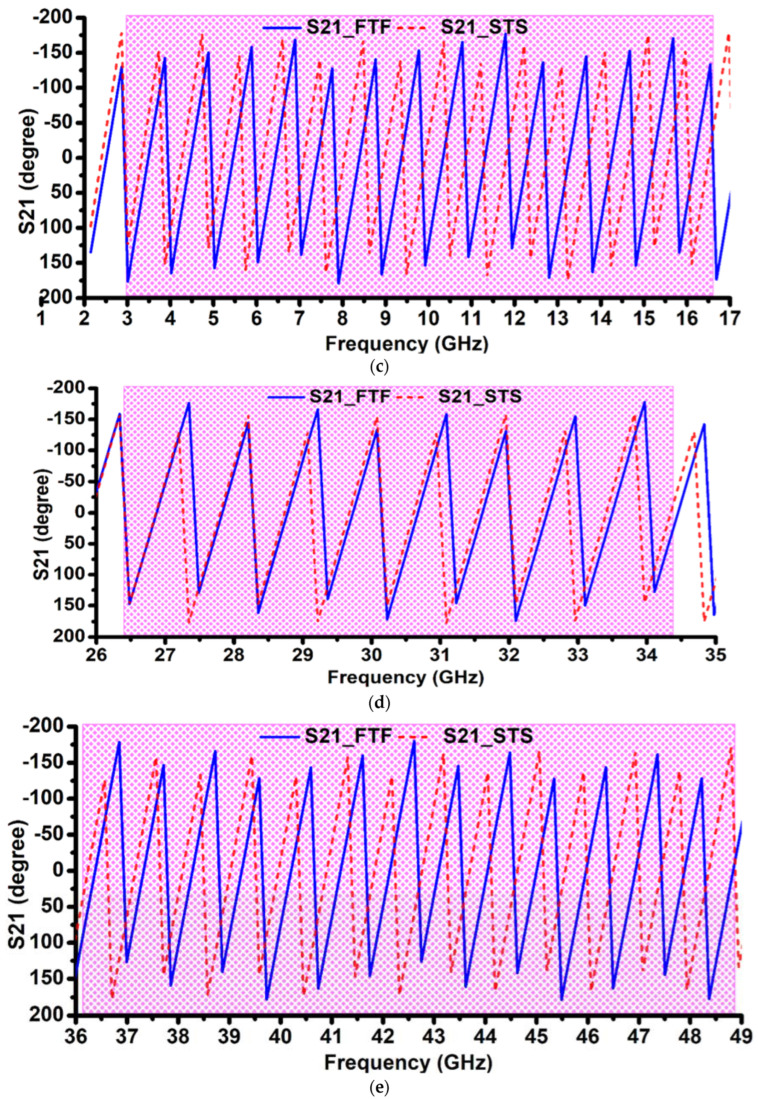
Simulated results at the different layout: (**a**) Group delay, (**b**) S21 (dB), (**c**) S21 (degree) over OFB1, (**d**) S21 (degree) over OFB2, and (**e**) S21 (degree) over OFB3.

Figure 12 shows the group delay (GD), magnitude of the S21, and phase of S21. From Figure 12a,b, it is clear that the group delay lies between 1010 and 1085 ps; however, the S21 dB was below −75 dB over bandwidths OFB1, OFB2, and OFB3. The S21 phase is shown in Figure 12c–e, showing the linear features within all the desired wideband operations in both FTF and STS layouts.

In order to discuss the time-domain analysis, the fidelity factor of antenna and system fidelity factor play a crucial role. The antenna fidelity factor (FF) is obtained by calculating the cross-correlation of the radiated E-field and the input signal; however, the system fidelity factor (SFF) is calculated by the cross-correlation between the transmitted pulse and the received pulse. The simulated value of FF and SFF are calculated in two different orientations (face to face and side by side), by keeping the proposed antenna 300 mm apart. Here the Gaussian sine pulse is given as input to the Tx port in order to find the FF and SFF. The obtained value of FF and SFF is presented in Table 2.

The antenna’s compact dimensions impact its transient behavior, and the level of pulse distortion is considered acceptable (SFF > 0.5 [54,55]), which is suitable for use in impulse-type UWB systems.

Table 3 indicates the recently reported literature comparison, in which the literature [8,9,13] covers the WLAN band and only [8] covers the n77/n78/n79 5G NR band. Here the proposed MIMO covers n77/n78/n79 5G NR band, WLAN, Wi-Fi, and wide band mm-Wave. None of the reported antennas in Table 1 calculates the CC, which is essential for investigating MIMO performance. The proposed MIMO system computed the CC in operating bands, which is quite suitable for investigated bands.

## 5. Conclusions

The proposed design operates on three wide bands that cover bands 3–15.5 GHz and two mm-wave bands (i.e., 26.4–34.3 GHz and 36.1–48.9 GHz). The minimum port isolation and ECC over the bands 3–15.5 GHz, 26.4–34.3 GHz, and 36.1–48.9 GHz are observed as (15.1 dB, 0.11), (22 dB, 0.035) and (22.37 dB, 0.04), respectively. The diversity performance of the MIMO antenna is performed by analyzing diversity gain, average efficiency, total interference ratio, channel capacity loss, and ergodic channel capacity. The MEG and TARC in all operating bands (i.e., 3–15.5 GHz, 26.4–34.3 GHz, and 36.1–48.9 GHz) are less than 0.2 dB and −4.1 dB, respectively. The CCL in all operating bands is less than 0.3 bps/Hz and the range of CC in operating bands 3–15.5 GHz, 26.4–34.3 GHz, and 36.1–48.9 GHz are 12.13–19.13 bps/Hz, 16.55–19.18 bps/Hz, and 16.92–18.44 bps/Hz, respectively. The simulated time-domain analysis in two different orientations, which are face to face (FTF) and side to side (STS), are studied. In both orientations, the group delays (GD) lie between 1010 and 1085 ps, and the S21 magnitude is less than −75 dB in all operating bands. The S21 phase provides linear characterization in all operating bands. The simulated values of FF and SFF are (0.9359, 0.7584) and (0.9477, 0.7583) in FTF and STS orientations, respectively. In all operating bands, the measured values provide good agreement with respect to the simulation ones, which indicates a good candidate for operation in all investigated bands.

## Figures and Tables

**Table 1:** Optimized lumped component values.

C1	C2	C3	C4	C5	C6	C7	C8	C9	C10	C11	C12
3.952	1.098	0.77	0.001	0.517	0.53	1.247	0.68	2.05	1.575	1.09	3.78
L1	L2	L3	L4	L5	L6	L7	L8	L9	L10	L11	L12
0.25	0.07	0.09	0.558	0.299	0.01	0.01	0.06	0.01	0.01	0.01	0.01
R1	R2	R3	R4	R5	R6	R7	R8	C1-C12 in pF, L1-L12 in nH, and R1-R8 in ohm			
48.79	112.8	38.62	90.25	38.62	80.67	45.64	26.74				

**Table 2:** Simulated value of FF and SFF.

Orientation	FF	SFF
face to face	0.9359	0.7584
side by side	0.9477	0.7583

**Table 3:** Comparison table of recently reported articles.

Ref	Bands		No. of Ports	Antenna Size/Substrate	Peak Gain	Peak Efficiency (%)	Isolation (dB)	ECC	TARC	CC (at −20 dB SNR) [bps/Hz]
[6]	dual band	1.87–2.53 GHz (6 dB)	2	0.44 × 0.73 × 0.007$\lambda_{0}^{3}$ (-)	4 dBi	75	15	0.18	-	-
		26–28.4 GHz (10 dB)			8 dBi	83	25	-	-	-
[7]	triple band	0.74–0.96 GHz (10 dB)	4	0.19 × 0.34 × 0.002$\lambda_{0}^{3}$ (Nelco N9000)	8 dBi	80	13	-	-	-
		1.7–2.2 GHz (10 dB)	1		-	-	40	-	-	-
		22–31 GHz (10 dB)			-	-	40	-	-	-
[8]	triple band	2.4–5.1 GHz (10 dB)	8	1.3 × 1.3 × 0.006$\lambda_{0}^{3}$ (RT/duroid 5880)	5 dBi	70	16	0.16	-	-
		5.1–5.6 GHz (10 dB)			5 dBi	70	16	0.16	-	-
		23–30 GHz (10 dB)			11 dBi	70	16	0.16	-	-
[9]	triple band	2.38–4.13 GHz (10 dB)	4	0.87 × 0.87 × 0.006$\lambda_{0}^{3}$ (RO5880)	3.47 dBi	93.58	>22	0.284	<−10 dB	-
		5.04–6.12 GHz (10 dB)			5.64 dBi	94.64	>22	-	<−10 dB	-
		22.28–29.28 GHz (10 dB)	4		11.4 dBi	93.26	>22	-	<−10 dB	-
[10]	dual band	2.05–2.7 GHz (10 dB)	4	0.55 × 0.47 × 0.003$\lambda_{0}^{3}$ (Rogers 5880)	4.5 dBi	70	20	0.01	-	-
		23–29 GHz (10 dB)			12.5 dBi	95	20	0.01	-	-
[11]	triple band	3.3–3.75 GHz (10 dB)	8	1.85 × 0.82 × 0.006$\lambda_{0}^{3}$ (Rogers RT/duroid 5880)	4 dBi	45	15	0.3 (AVG)	-	-
		27.15–28.77 GHz (10 dB)			6 dBi	60	>25	<<0.3 (AVG)	-	-
		37.59–38.49 GHz (10 dB)			6 dBi	60	>25	<<0.3 (AVG)	-	-
[13]	quad band	2.54–2.64 GHz (10 dB)	4	0.33 × 0.33 × 0.014$\lambda_{0}^{3}$ (FR4)	1 dBi	-	>21.5	<0.012	-	-
		3.4–3.62 GHz (10 dB)			3 dBi	-	>21.5	<0.012	-	-
		4.4–5.28 GHz (10 dB)			3.5 dBi	-	>21.5	<0.012	-	-
		5.6–6 GHz (10 dB)			1.6 dBi	-	>21.5	<0.012	-	-
[14]	dual band	2.99–3.61 GHz (10 dB)	2	0.68 × 0.28 × 0.017$\lambda_{0}^{3}$ (Rogers RO4003)	3.14 dB	80.24 (RAD)	>25	<0.002	<−25 dB	-
		4.53–4.92 GHz (10 dB)			3.84 dB	84.64 (RAD)	>16	<0.002	<−20 dB	-
[56]	single band	5.37–11.0 GHz (10 dB)	4	1.364 × 1.364 × 0.013$\lambda_{0}^{3}$ (FR4)	5.6 dB	-	>21	<0.003	0.0001 dB	
[57]	quad band	1.21–1.24 GHz (10 dB)	4	0.246 × 0.246 × 0.0065$\lambda_{0}^{3}$ (FR4)	1.73 dBi	-	>22	<0.003	-	-
		2.42–2.5 GHz (10 dB)			4.83 dBi	-	>22	<0.003	-	-
		3.4–3.6 GHz (10 dB)			7.62 dBi	-	>22	<0.003	-	-
		4.8–5.1 GHz (10 dB)			9.81 dBi	-	>22	<0.003	-	-
[58]	single band	25–31.6 GHz (10 dB)	2	1.01 × 1.85 × 0.154$\lambda_{0}^{3}$ (FR4)	4.8 dBi	-	>25	<0.01	-	-
[59]	single band	3.4–3.6 GHz (6 dB)	8	0.233 × 0.145 × 0.0093$\lambda_{0}^{3}$ (FR4)	2.87 dBi	65	>12	<0.2	-	38
**This work**	**triple band**	**3–17 GHz (10 dB)**	4	**1.057 × 1.057 × 0.053** $\lambda_{0}^{3}$ **(FR4)**	**3.03 dB**	**56.7**	**>15.1**	**<0.21**	**<−5 dB**	**12.1–19.1**
		**25.3–35.1 GHz (10 dB)**			**5.87 dB**	**58.8**	**>22**	**<0.034**	**<−7.4 dB**	**16.6–19.2**
		**35.5–49.4 GHz (10 dB)**			**5.92 dB**	**52.5**	**>22.37**	**<0.04**	**<−6.9 dB**	**16.9–18.4**

$\lambda_{0}^{3}$→ free space wavelength (mm) at lowest operating frequency band **RAD**→ Radiation efficiency **AVG**→ Average ECC over entire operating band.

## References

[1] Yang B., Yu Z., Lan J., Zhang R., Zhou J., Hong W. (2018). Digital Beamforming-Based Massive MIMO Transceiver for 5G Millimeter-Wave Communications. IEEE Trans. Microw. Theory Tech..

[2] Bjornson E., Van der Perre L., Buzzi S., Larsson E.G. (2019). Massive MIMO in sub-6 GHz and mmWave: Physical, practical, and use-case differences. IEEE Wirel. Commun..

[3] Jehangir S.S., Sharawi M.S. (2017). A Single Layer Semi-Ring Slot Yagi-Like MIMO Antenna System with High Front-to-Back Ratio. IEEE Trans. Antennas Propag..

[4] Sharawi M.S., Ikram M., Shamim A. (2017). A Two Concentric Slot Loop Based Connected Array MIMO Antenna System for 4G/5G Terminals. IEEE Trans. Antennas Propag..

[5] Ikram M., Hussain R., Sharawi M.S. (2017). 4G/5G antenna system with dual function planar connected array. IET Microwaves, Antennas Propag..

[6] Hussain R., Alreshaid A.T., Podilchak S.K., Sharawi M.S. (2017). Compact 4G MIMO antenna integrated with a 5G array for current and future mobile handsets. IET Microwaves, Antennas Propag..

[7] Taheri M.M.S., Abdipour A., Zhang S., Pedersen G.F. (2019). Integrated Millimeter-Wave Wideband End-Fire 5G Beam Steerable Array and Low-Frequency 4G LTE Antenna in Mobile Terminals. IEEE Trans. Veh. Technol..

[8] Ikram M., Nguyen-Trong N., Abbosh A. (2019). Multiband MIMO microwave and millimeter antenna system employing dual-function tapered slot structure. IEEE Trans. Antennas Propag..

[9] Jabeen S., Khan Q.U. (2022). An integrated MIMO antenna design for Sub-6 GHz & millimeter-wave applications with high isolation. AEU—Int. J. Electron. Commun..

[10] Ikram M., Al Abbas E., Nguyen-Trong N., Sayidmarie K.H., Abbosh A. (2019). Integrated Frequency-Reconfigurable Slot Antenna and Connected Slot Antenna Array for 4G and 5G Mobile Handsets. IEEE Trans. Antennas Propag..

[11] Islam S., Zada M., Yoo H. (2021). Low-Pass Filter Based Integrated 5G Smartphone Antenna for Sub-6-GHz and mm-Wave Bands. IEEE Trans. Antennas Propag..

[12] Zada M., Shah I.A., Yoo H. (2021). Integration of Sub-6-GHz and mm-Wave Bands with a Large Frequency Ratio for Future 5G MIMO Applications. IEEE Access.

[13] Liu X., Zhang J., Xi H., Yang X., Sun L., Gan L. (2022). A compact four-band high-isolation quad-port MIMO antenna for 5G and WLAN applications. AEU—Int. J. Electron. Commun..

[14] Sharma P., Tiwari R.N., Singh P., Kanaujia B.K. (2022). Dual-band trident shaped MIMO antenna with novel ground plane for 5G applications. AEU—Int. J. Electron. Commun..

[15] Niu Z., Zhang H., Chen Q., Zhong T. (2019). Isolation Enhancement for 1 × 3 Closely Spaced E-Plane Patch Antenna Array Using Defect Ground Structure and Metal-Vias. IEEE Access.

[16] Tan X., Wang W., Wu Y., Liu Y., Kishk A.A. (2019). Enhancing Isolation in Dual-Band Meander-Line Multiple Antenna by Employing Split EBG Structure. IEEE Trans. Antennas Propag..

[17] Farahat A.E., Hussein K.F.A. (2022). Dual-Band (28/38 GHz) Wideband MIMO Antenna for 5G Mobile Applications. IEEE Access.

[18] Sabek A.R., Ali W.A.E., Ibrahim A.A. (2022). Minimally Coupled Two-Element MIMO Antenna with Dual Band (28/38 GHz) for 5G Wireless Communications. J. Infrared, Millimeter, Terahertz Waves.

[19] Hasan N., Bashir S., Chu S. (2019). Dual band omnidirectional millimeter wave antenna for 5G communications. J. Electromagn. Waves Appl..

[20] Sharma M., Awasthi Y.K., Singh H., Kumar R., Kumari S. (2016). Design of Compact Flower Shape Dual Notched-Band Monopole Antenna for Extended UWB Wireless Applications. J. RF Eng. Telecommun..

[21] Addepalli T., Kamili J.B., Bandi K.K., Nella A., Sharma M. (2022). Lotus flower-shaped 4/8-element MIMO antenna for 5G n77 and n78 band applications. J. Electromagn. Waves Appl..

[22] Khandelwal M.K., Kanaujia B.K., Kumar S. (2017). Defected Ground Structure: Fundamentals, Analysis, and Applications in Modern Wireless Trends. Int. J. Antennas Propag..

[23] Keysight Inc (2022). Advanced Design System (ADS). Santa Rosa, CA, USA. https://www.keysight.com/.

[24] Jangid S., Rama V.S.B. (2014). An Equivalent Circuit Modeling of UWB Patch Antenna with Band Notched Characteristics. Eur. J. Adv. Eng. Technol..

[25] Koohestani M., Azadi-Tinat N., Skrivervik A.K. (2023). Compact slit-loaded ACS-Fed monopole antenna for Bluetooth and UWB systems with WLAN band-stop capability. IEEE Access.

[26] Wu D., Wang J., Cai Y., Guizani M. (2015). Millimeter-wave multimedia communications: Challenges, methodology, and applications. IEEE Commun. Mag..

[27] Ford R., Zhang M., Mezzavilla M., Dutta S., Rangan S., Zorzi M. (2017). Achieving ultra-low latency in 5G millimeter wave cellular networks. IEEE Commun. Mag..

[28] Liu X., Cai Z. (2010). Advanced Obstacles Detection and Tracking by Fusing Millimeter Wave Radar and Image Sensor Data.

[29] Bevacqua M.T., Di Meo S., Crocco L., Isernia T., Pasian M. (2021). Millimeter-waves breast cancer imaging via inverse scattering techniques. IEEE J. Electromagn. Rf Microw. Med. Biol..

[30] Johnson J.E., Shay O., Kim C., Liao C. (2019). Wearable millimeter-wave device for contactless measurement of arterial pulses. IEEE Trans. Biomed. Circuits Syst..

[31] Harvey J.F., Steer M.B., Rappaport T.S. (2019). Exploiting High Millimeter Wave Bands for Military Communications, Applications, and Design. IEEE Access.

[32] Xiao Z., Zhu L., Liu Y., Yi P., Zhang R., Xia X.-G., Schober R. (2021). A Survey on millimeter-wave beamforming enabled UAV communications and networking. IEEE Commun. Surv. Tutorials.

[33] Mazaheri M.H., Ameli S., Abedi A., Abari O. A millimeter wave network for billions of things. Proceedings of the ACM Special Interest Group on Data Communication.

[34] Sharaf M.H., Zaki A.I., Hamad R.K., Omar M.M. (2020). A novel dual-band (38/60 GHz) patch antenna for 5G mobile handsets. Sensors.

[35] Peng M., Zhao A. (2018). High performance 5G millimeter-wave antenna array for 37–40 GHz mobile application. 2018 International Workshop on Antenna Technology (iWAT).

[36] Park J., Choi D., Hong W. GHz vertically-polarized end-fire 5G antenna array featuring electrically small profile. Proceedings of the 2018 IEEE International Symposium on Antennas and Propagation & USNC/URSI National Radio Science Meeting.

[37] Malekar R.R., Shevada L.K., Raut H.D., Dixit A.S., Kumar S. (2020). MIMO antenna for fifth generation mm-wave applications: A bibliometric survey. Libr. Philos. Pract..

[38] Ali W., Das S., Medkour H., Lakrit S. (2021). Planar dual-band 27/39 GHz millimeter-wave MIMO antenna for 5G applications. Microsyst. Technol..

[39] Khalid M., Iffat Naqvi S., Hussain N., Rahman M., Fawad, Mirjavadi S.S., Khan M.J., Amin Y. (2020). 4-Port MIMO antenna with defected ground structure for 5G millimeter wave applications. Electronics.

[40] Yoon N., Seo C. (2017). A 28-GHz wideband 2× 2 U-slot patch array antenna. J. Electromagn. Eng. Sci..

[41] Abbas M.A., Allam A., Gaafar A., Elhennawy H.M., Sree M.F.A. (2023). Compact UWB MIMO antenna for 5G millimeter-wave applications. Sensors.

[42] Shah S.T., Shakir S., Durani M.H., Ahmed U., Bilal M. December. Miniaturized four port MIMO antenna system for 5G mm-wave applications. Proceedings of the 2021 1st International Conference on Microwave, Antennas & Circuits (ICMAC).

[43] Joseph J., Let G.S., Pratap C.B., Winston J.J. (2023). A miniaturized uniplanar MIMO antenna for n79/n46/millimeter-wave applications. Int. J. Commun. Syst..

[44] Wang F., Duan Z., Wang X., Zhou Q., Gong Y. (2019). High isolation millimeter-wave wideband MIMO antenna for 5G communication. Int. J. Antennas Propag..

[45] Sharawi M.S., Hassan A.T., Khan M.U. (2017). Correlation coefficient calculations for MIMO antenna systems: A comparative study. Int. J. Microw. Wirel. Technol..

[46] Singh A.K., Mahto S.K., Kumar P., Mistri R.K., Sinha R. (2022). Reconfigurable circular patch MIMO antenna for 5G (sub-6 GHz) and WLAN applications. Int. J. Commun. Syst..

[47] Sahu N.K., Das G., Gangwar R.K. (2018). Dual polarized triple-band dielectric resonator based hybrid MIMO antenna for WLAN/WiMAX applications. Microw. Opt. Technol. Lett..

[48] Fritz-Andrade E., Jardon-Aguilar H., Tirado-Mendez J.A. (2019). The correct application of total active reflection coefficient to evaluate MIMO antenna systems and its generalization to N ports. Int. J. RF Microw. Comput. Eng..

[49] Guo J., Cui L., Li C., Sun B. (2018). Side-Edge Frame Printed Eight-Port Dual-Band Antenna Array for 5G Smartphone Applications. IEEE Trans. Antennas Propag..

[50] Mistri R.K., Singh A.K., Mahto S.K., Sinha R. (2023). Quad element millimetre-wave MIMO antenna for 5G communication. J. Electromagn. Waves Appl..

[51] Yun J.X., Vaughan R.G. (2012). Multiple Element Antenna Efficiency and its Impact on Diversity and Capacity. IEEE Trans. Antennas Propag..

[52] Mistri R.K., Mahto S.K., Sinha R. (2022). Dual band 8 × 8 MIMO antenna system for DCS 1800 and 5G mobile applications. Int. J. Commun. Syst..

[53] Zou H., Li Y., Sim C., Yang G. (2018). D esign of 8 × 8 dual-band MIMO antenna array for 5 G smartphone applications. Int. J. RF Microw. Comput. Eng..

[54] Quintero G., Zurcher J.F., Skrivervik A.K. (2011). System fidelity factor: A new method for comparing UWB antennas. IEEE Trans. Antennas Propag..

[55] Koohestani M., Zürcher J.F., Moreira A.A., Skrivervik A.K. (2014). A novel, low-profile, vertically-polarized UWB antenna for WBAN. IEEE Trans. Antennas Propag..

[56] Kollipara V., Peddakrishna S. (2022). Quad-Port Circularly Polarized MIMO Antenna with Wide Axial Ratio. Sensors.

[57] Nej S., Ghosh A., Ahmad S., Ghaffar A., Hussein M. (2022). Compact Quad Band MIMO Antenna Design with Enhanced Gain for Wireless Communications. Sensors.

[58] Ravi K.C., Kumar J. (2022). Miniaturized Parasitic Loaded High-Isolation MIMO Antenna for 5G Applications. Sensors.

[59] Kiani S.H., Altaf A., Anjum M.R., Afridi S., Arain Z.A., Anwar S., Khan S., Alibakhshikenari M., Lalbakhsh A., Khan M.A. (2021). MIMO Antenna System for Modern 5G Handheld Devices with Healthcare and High Rate Delivery. Sensors.

